# The Role of Splicing Machinery as a Promising Potential Therapeutic Target for Pituitary Neuroendocrine Tumors (PitNETs)

**DOI:** 10.3390/cancers18162544

**Published:** 2026-08-07

**Authors:** Federica Mangili, Donatella Treppiedi, Erika Peverelli, Giovanna Mantovani

**Affiliations:** 1 Endocrinology Unit, Fondazione IRCCS Ca’ Granda Ospedale Maggiore Policlinico, 20122 Milan, Italy; federica.mangili@policlinico.mi.it (F.M.); donatella.treppiedi@policlinico.mi.it (D.T.); giovanna.mantovani@unimi.it (G.M.); 2 University of Milan, Department of Clinical Sciences and Community Health, Department of Excellence 2023–2027, 20122 Milan, Italy

**Keywords:** pituitary neuroendocrine tumors (PitNETs), drug resistance, splicing machinery alteration, splicing factors

## Abstract

Pituitary neuroendocrine tumors (PitNETs) represent one of the most common intracranial lesions. The pharmacological approach to PitNETs is mainly based on the use of somatostatin (SS) receptor ligands (SRLs) and dopamine agonists (DAs), although resistance or poor efficacy occurs in a percentage of patients. SS receptors (SSTs) and dopamine receptor type 2 (DRD2) represent the principal targets of SRLs and DAs, respectively, in PitNET therapy. Over the years, SSTs and DRD2-related activated pathways have been characterized. Different factors that might play a role in affecting intracellular signaling transduction have been studied, including splicing machinery and its related factors. A severe dysregulation of splicing machinery components in all PitNET subtypes compared to normal pituitary was observed, and possible alterations that might underlie pharmacological resistance or intracellular pathway alterations were investigated. The detection of these alterations presented the opportunity to investigate small molecules that regulate splicing components as an alternative approach for PitNETs treatment. A systematic literature search was conducted in the PubMed database and supplemented by screening the references of the identified articles. A narrative synthesis of the research findings was provided, including relevant studies from 2015 up to date. It aimed to summarize the emerging role of splicing machinery, focusing on different splicing factors shown to be altered in different PitNET subtypes that might affect responsiveness to pharmacological treatment. Different ways to target splicing machinery and its mechanism of action have been described, with the goal of developing novel therapies for PitNETs.

## Simple Summary

Pituitary neuroendocrine tumors (PitNETs) are a heterogeneous group of lesions that represent 10–25% of all intracranial neoplasms and are the most common pathology of the pituitary gland. Even if they are generally benign, PitNETs can cause visual field deficits, neurological manifestations, and endocrine syndromes. Nevertheless, resistance or poor efficacy to available pharmacological therapies occurs in a percentage of patients. The effects of splicing machinery and its associated factors on response to treatment were highlighted. Here, we aimed to summarize the role of splicing machinery dysregulation in the behavior and responsiveness of PitNETs to pharmacological treatment. Additionally, we propose potential novel therapeutic approaches targeting splicing machinery to broaden the pharmacological spectrum for PitNETs treatment.

## 1. Pituitary Neuroendocrine Tumors (PitNETs)

PitNETs are a heterogeneous group of lesions that represent 10–25% of all intracranial neoplasms and are the most common pathology of the pituitary gland. They can be classified according to their hormonal secretion in functioning (growth (GH), adrenocorticotropic (ACTH), prolactin (PRL), thyroid-stimulating (TSH), luteinizing and follicle-stimulating hormones (FSH/LH), secreting-PitNET) or non-functioning (NF-PitNETs). Even if they are generally benign, PitNETs can cause visual field deficits and neurological manifestations due to “mass effects” and/or endocrine syndromes. Moreover, PitNETs frequently cause mass-effect symptoms through compression of the optic chiasm and/or invasion of adjacent structures, including the cavernous sinus and infrasphenoidal bone. They may also be complicated by pituitary apoplexy, an acute hemorrhagic or ischemic event occurring within the tumor [1,2].

PitNETs’ classification criteria are lineage-restricted and based on pituitary transcription factors, according to the 5th WHO classification [3]. Pharmacological therapies with DAs and SRLs are currently approved for a subset of patients with secreting PitNETs. Specifically, DA treatment represents the first-line medical approach for PRL-PitNETs, whereas SRLs (such as lanreotide, pasireotide, and octreotide) represent a pharmacological chance for patients with GH- and ACTH-PitNETs; however, a percentage of patients experience recurrence or resistance, limiting their efficacy. The targets of SRLs and DAs are SSTs and DRD2, respectively, based on their ability to significantly inhibit cell proliferation and normalize circulating hormones [4].

## 2. RNA Alternative Splicing and Its Role in Cancer

Alternative splicing (AS) is a finely regulated post-transcriptional process that enables a majority of genes (>95%) to produce variant mature mRNA isoforms from a single precursor (pre-mRNA) [5,6]. The core splicing machinery, known as the spliceosome, is a ribonucleoprotein complex that removes introns and joins exons to generate mature mRNA. Moreover, many regulatory proteins are implicated in correctly coordinating and catalyzing the reaction. Among the non-spliceosomal nuclear RNA-binding proteins, the serine/arginine (SR) splicing factors (SFs) (SRSFs) and the heterogeneous nuclear ribonucleoprotein (hnRNP) families are the most studied. SRSFs’ localization and activity are regulated by specific kinases (SRPK1/2 and the CDC2-like kinases CLK1-4) able to phosphorylate the serine residues in their SR domains [7], allowing SRSFs to interact with other SFs or RNA, as reviewed in a study by Urbanski and co-authors [8]. Multiple mechanisms of AS have been described, including exon skipping, mutually exclusive exon selection, exon scrambling, alternative 3′ or 5′ splice sites, intron retention, alternative promoter, and alternative polyadenylation.

Due to the complexity of the splicing reaction, incorrect removal of introns and/or exons, as well as altered expression of splicing machinery components, may result in aberrant splicing variants with the potential to initiate a malignant phenotype [9]. Altogether, these altered splice-site signals may create alternative splice variants of tumor suppressor genes such as p53, BRCA1, APC, and others [10].

In addition, mutations in key splicing regions of genes regulating cell cycle progression, signal transduction, angiogenesis, motility, invasion, and apoptosis may contribute to determining the disease state [10] across different types of tumors.

A large number of studies have demonstrated the existence of splicing trans-alterations in cancer, involving deregulated expression of various components of the spliceosome machinery or somatic mutations occurring in their genes [8,11].

Regarding PitNETs, the mechanisms underlying their tumorigenesis remain debated, as relatively few classical somatic mutations have been found among secreting and NF-PitNETs [12]. Recently, several splicing cis- and trans-alterations have been identified in all PitNET subtypes and will be further discussed in the present review.

## 3. Splicing Machinery Dysregulation in PitNETs

### 3.1. Deregulated Expression of Splicing Machinery Components

Functional alterations of the splicing-regulatory machinery, which compromise the physiological splicing process of a wide range of genes, have recently been found among all PitNET subtypes [13,14]. In these studies, the mRNA expression of core spliceosome components and SRSFs was analyzed in large cohorts encompassing all PitNET subtypes, using non-pathological pituitary glands as a control group. Analyses were performed using the dynamic quantitative real-time PCR (qPCR) microfluidic array technique [13] or bulk RNA sequencing, and were further validated by full-length single-cell RNA sequencing, allowing more precise identification of tumor cell populations for each subtype [14].

In detail, the innovative study conducted by Huang and co-authors [14] explored the transcriptomic splicing features in 264 tumor samples from all PitNET subtypes, with validation by an independent cohort of 180 PitNETs. PitNETs splicing heterogeneity at single-cell resolution was delineated, confirming bulk findings and refining splicing dysregulation across different PitNET cell types. The analysis revealed widespread dysregulation of RNA-binding motif proteins (RBPs), indirectly confirming the splicing disruptions in PitNETs, even if the underlying reasons for such changes remain unknown.

In particular, splicing feature analysis has identified the silent corticotrophs subtype and a distinct TPIT lineage subtype; the latter is associated with worse clinical outcomes and increased splicing abnormalities driven by altered expression of RBP Epithelial Splicing Regulatory Protein 1 (ESRP1) [14]. ESRP1, a key epithelial splicing regulatory factor, plays a key role in the epithelial–mesenchymal transition (EMT) process, highlighting its strong association with PitNETs progression. Two different subgroups have been distinguished: the first is characterized by lower levels of splicing alterations, showing similarities to functional ACTH in terms of biological characteristics and clinical outcomes, indicating a potential transitional state between functional and silent ACTH. The second, displaying higher levels of splicing alterations, exhibited poorer clinical outcomes with higher recurrence rates, suggesting a more aggressive phenotype [14].

Additionally, a study by Vázquez-Borrego and co-authors highlighted a tumor subtype-dependent pattern of splicing machinery dysregulation, with NF-PitNETs showing a robust downregulation of most of the analyzed genes within the Spanish cohort (Table 1). Conversely, secreting PitNETs showed upregulation of many components [13]. Briefly, in GH-PitNETs, RBM3 and ribonucleoprotein PTB-binding 1 (RAVER1) were the SFs that had a higher capacity to discriminate between tumoral and control groups. RBM3, like other RBPs, identifies secondary or tertiary structures in RNA and influences the assembly of the spliceosome complex, playing a crucial role in cell differentiation. RAVER1 shuttles between the nucleus and cytoplasm, binding to microfilaments and linking gene expression to structural function. In GH-PitNETs, RAVER1 expression levels were associated with a lower rate of cavernous sinus invasion and extrasellar growth and directly correlated with the expression of the aberrant splicing variant In1-ghrelin [13]. High levels of SRSF6 were found in GH-PitNETs of patients pre-treated with SRLs; according to the authors’ analysis of patients’ clinicopathological characteristics, they were markedly linked to a lower rate of cavernous sinus invasion [13]. In ACTH-PitNETs, Mago-nashi homolog (MAGOH), K homology domain-containing protein, RNA-binding protein, and signal transduction-associated protein 1 (KHDRBS1) were strongly overexpressed. MAGOH is a key component of the exon junction complex and participates in regulating physiological processes such as cell cycle regulation [15,16] and apoptosis [17,18,19]. In ACTH-PitNETs, MAGOH overexpression was associated with less chiasmatic compression and better treatment responsiveness [13]. MAGOH was the only upregulated SF, together with the SR repetitive matrix protein 4 (SRRM4), in NF-PitNETs (Table 1). SRRM4 is a key regulator of micro-exons incorporation into mature mRNA, significantly expanding the functional protein repertoire in the nervous system. In NF-PitNETs, its overexpression was associated with higher chiasmatic compression [13]. Moreover, a common pattern of dysregulation of two minor spliceosome components (RNU11 and RNU6ATAC) and two SFs (SRSF1 and SRRM4) in most PitNETs was detected, irrespective of their subtype. SRSF1 is positively correlated with the oncogenic splicing variant SST5TMD4 in GH-, ACTH-, and NF-PitNETs, and both RNU11 and RNU6ATAC are correlated with SST5TMD4 in GH-PitNETs [13] (Table 1).

In a Chinese cohort, a profoundly altered pattern in the RBP family that specifically governs subtype-selective splicing disorders in each PitNET lineage has been found [14]. In line with previous studies [20,21,22,23], a tightly connected regulatory network between the main altered RBPs and AS events was generated, and genes with the most splicing variants showed enrichment in various pathways, such as GTPase regulator activity, protein metabolic process, nitrogen compound transport, cell junction, and developmental process pathways. Notably, a significant increase in ESRP1 expression levels was found in ACTH-secreting cells compared to non-functional cells [14]. Patients with lower ESRP1 expression in the silent group were characterized by a worse progression-free survival (PFS). ESRP1-regulated splicing events (in ENAH, ARFGAP1, and VTI1A genes) also exhibited a marked impact on PFS. Indeed, these subtype-specific events were associated with larger tumor size, augmented invasion, and higher Ki67, suggesting a more aggressive phenotype and highlighting the clinical significance of specific splicing characteristics as biomarkers for disease progression. Moreover, a correlation between ESRP1 expression and pre-operative ACTH levels was observed, indicating that measuring ESRP1 levels could provide additional guidance for treatment selection [14].

Another interesting study conducted by Wang and co-authors [24] unveiled an overexpression of the SF FUS in PIT-1-positive PitNETs compared to normal pituitary, associating it with a significantly increased risk of postoperative hypopituitarism. Specifically, the authors showed FUS promotes the inclusion of exon 3 in MDM2 pre-mRNA, thereby increasing MDM2 levels and suppressing p53 activity. These findings identify FUS as an essential oncogenic SF with therapeutic potential in PIT1-lineage PitNETs [24].

### 3.2. Mutation in the Splicing Factor 3B Subunit 1 (SF3B1)

In addition, altered expression levels of spliceosome machinery components and somatic mutations in critical genes, including SF3B subunit 1 (SF3B1), U2AF1, and ZRSR2, which are all components of the U2 snRNP complex, as well as the SR protein SRSF2, have been found in different types of cancers [8,24]. In PitNETs, the discovery and characterization of the SF3B1 mutation strongly contributed to deciphering PRL-PitNETs tumorigenesis. SF3B1 is a crucial component essential for the pre-RNA process, encoding a core component of the U2 small nuclear ribonucleoprotein (snRNP) required for the splicing of most introns, being involved in the recognition of the branch-site for early-stage spliceosome assembly [25,26]. SF3B1 comprises an intrinsically unstructured N-terminal domain (NTD) and a C-terminal domain containing 20 HEAT (Huntingtin, Elongation factor 3, PR65/A, TOR) repeats, which are tandem repeat structural motifs of two alpha helices linked by a short loop, forming a right-handed superhelix [27].

Gain-of-function mutations in this gene can lead to abnormal splicing and subsequently affect the regulation of gene expression [26]. Specifically, SF3B1 has an important oncogenic role in the pathogenesis and development of many tumors and has been found to be mutated in both hematological and solid ones, such as breast carcinoma, skin cutaneous or uveal melanomas, pancreatic ductal adenocarcinoma, or prostate cancer [28,29,30,31]. The somatic mutation affecting SF3B1, SF3B1^R625H^, has been described in 19.8% of patients with PRL-PitNETs [32]. This mutation resulted in a gain-of-function of the estrogen-related receptor gamma (ESRRG) through alternative splicing and has been associated in patients with enhanced PRL secretion, shorter PFS, increased cell proliferation, and decreased cell apoptosis in vitro [32]. Moreover, it has also been demonstrated that rat pituitary GH3 cells expressing SF3B1^R625H^ exhibited higher PRL levels and invasive behavior, induced by phosphatidylinositol 3-kinase (PI3K)/protein-kinase B pathway activation and downregulation of Discs large 1 (DLG1) [33]. Another recent study by Simon and co-authors reported SF3B1 variants in 7 out of 282 PRL-PitNET samples derived from eight different European centers. In particular, they detected the SF3B1^R625H^ mutation in four samples, three of which were metastatic PRL-PitNETs, and unveiled a previously undescribed variant, c.1873C > T (p.Arg625Cys), in three aggressive tumors [34], suggesting that SF3B1 somatic mutations in PRL-PitNETs are uncommon in European centers and are associated with aggressive tumor behavior. The No R625H SF3B1 germline mutation has been identified in patients with macroprolactinomas [35].

Interestingly, a recent work also analyzed SF3B1^R625H^ prevalence in a cohort of 127 patients with PRL-PitNETs from 17 different hospitals, using both digital and digital droplet PCR, correlating it with patients’ main clinicopathological features. The somatic SF3B1^R625H^ mutation was detected in 17% of tumors, with a higher fractional abundance of the mutation in males and in patients who underwent transcranial surgery. Mutation-positive patients were associated with younger age and significantly larger tumors, highlighting a potential role of SF3B1 in early tumorigenesis and tumor growth [36] (Table 1). Moreover, their findings suggest that mutation status did not significantly impact DA resistance in the analyzed cohort, although further studies are needed to better characterize the potential association between SF3B1 mutations and PRL-PitNETs’ clinicopathological features. Additionally, in vitro data demonstrated that SF3B1^R625H^ overexpression in tumoral lactotrophs resulted in an increase in both cell proliferation and migration, along with a reduction in DRD2 expression at both the protein and transcript levels [37].

**Table 1:** Altered splicing factors in different PitNET subtypes. The main altered (overexpressed, down regulated, altered, or mutated) factors in different PitNET subtypes, together with their biological effects, therapeutic potential, and clinical relevance, have been summed up.

	Altered Splicing Factor	PitNET Subtype	Biological Effects in PitNETs	Therapeutic Relevance	Associated Clinical Features in PitNETs	Reference
**REDUCED EXPRESSION**	**ESRP1**	ACTH-PitNETs	Regulates EMT; promotes tumor progression	biomarker for tumor progression	worse PFS, larger tumor size, augmented invasion, higher Ki67	[14]
	**RNU6ATAC**	NF-PitNETs	*nd*	*nd*	*nd*	[13]
	**RNU11**	PRL-, GH-, NF-PitNETs	*nd*	*nd*	*nd*	[13]
**OVEREXPRESSION**	**RAVER-1**	GH-, PRL-PitNETs	*nd*	potential tumor-suppressing factor	lower rate of cavernous sinus invasion and extrasellar growth	[13]
	**SRSF6**	PRL-, GH-PitNETs	*nd*	correlated with pre-treatment with SRLs	lower rate of cavernous sinus invasion	[13]
	**MAGOH**	All PitNETs subtypes	regulation of cell cycle and apoptosis	Potential predictor of treatment response; higher curation rate	less chiasmatic compression and better treatment responsiveness	[13]
	**SRRM4**	PRL-, GH-, NF-PitNETs	*nd*	*nd*	higher chiasmatic compression in NF-PitNETs	[13]
	**FUS**	PIT-1 positive PitNETs	suppression of p53 activity	essential oncogenic SF with therapeutic potential	increased risk of postoperative hypopituitarism	[24]
	**RBM3**	GH-, -PitNETs	*nd*	*nd*	*nd*	[13]
**DYSREGULATION**	**SRSF1**	GH-, NF-PitNETs	leads to a decrease in VEGF splicing isoform production; pro-mitotic, pro-angiogenic and pro-migratory effect in GH-PitNETs	potential therapeutic target	*nd*	[13]
**MUTATION**	**SF3B1 (R625H)**	PRL-PitNETs	PRL hypersecretion, enhanced cell proliferation, migration and invasion; reduced apoptosis	resistance to DAs treatment	worse PFS, younger age and significantly larger tumors	[32,33,34,35,36,37]

## 4. Clinically Relevant Aberrant Splicing Variants in PitNETs

Recent studies have indicated that splicing alterations in key genes (ESRRG, GHRHR, In1-ghrelin, IGF1R, EGFR, PTEN, and CCND1) involved in different signal transduction pathways are present in PitNETs [13,32,38]. Splicing transcript variants in the inhibitory SS and dopamine pathways have been reported as well, specifically in the SST5 and DRD2 genes. Splicing variants of some of these most studied genes are reviewed in detail and are summarized in Table 2.

### 4.1. Estrogen-Related Receptor Gamma (ESRRG)

Another gene whose splicing variant was highlighted as having a role in PitNETs is estrogen-related receptor gamma (ESRRG). ESRRG belongs to the ESRR family, whose biological functions are involved in estrogen-signaling pathways. Interestingly, Li and co-authors unveiled that the specific SF3B1^R625H^ mutation causes aberrant splicing of ESRRG, resulting in a gain-of-function that determines stronger binding of pituitary-specific positive transcription factor 1 (PIT-1), leading to excessive PRL secretion [32]. They showed that aberrant ESRRG is transcriptionally active and induces abnormal PRL transcription, hypothesizing that the higher PRL levels in SF3B1 mutant PRL-PitNETs are likely caused by this alteration. Moreover, data evaluation showed a gender preference in the mutated PRL-PitNETs subset with 24.34% of the total population of males versus 10.67% of females, differing from the historically observed female gender dominance of this PitNET subtype [39,40] (Table 2). The ESRRG-mediated mechanism of PRL secretion showed ER independence, supporting the observed gender distribution.

### 4.2. Growth Hormone Releasing Hormone Receptor (GHRHR)

Hypothalamic growth hormone-releasing hormone (GHRH) is well known to stimulate synthesis and secretion of GH, influencing multiple biological functions through the GHRH receptor (GHRHR), which is a G protein-coupled receptor (GPCR) [41]. Ligands binding to GHRHR activate the Gsα subunit of the associated G-protein complex, which stimulates adenylyl cyclase, resulting in the conversion of ATP to cAMP, which stimulates GH secretion [42] and mediates the proliferative actions of GHRH in somatotroph cells.

As with many genes, the GHRHR gene can undergo AS, generating multiple functional splicing variants that have yet to be identified in different cancer types [43], and the hypothesis that this might be induced by a hypoxic microenvironment in solid tumors, leading to enhanced glycolysis, has been proposed [44]. Both GHRH and GHRHR have been extensively investigated in different NETs, mainly for GHRH ectopic expression.

Among the four different GHRHR splicing variants that have been identified, the GHRHR splicing variant 1 (SV1) has been the most studied, since it is the most similar to full-length GHRHR. Specifically, GHRHR SV1 lacks a portion of the extracellular domain since the first three exons are replaced by a fragment of intron 3, leading to the replacement of the first 89 amino acids of GHRHR with a distinct 25-amino acid sequence [43]. GHRHR SV1 mitogenic activity was demonstrated [45,46]. Moreover, it was demonstrated that GHRHR SV1 was able to strongly activate the MAPK-ERK1/2 signaling pathway in HeLa cells [45], suggesting that this splicing variant may also have a role in the main pathways that regulate PitNETs intracellular signaling and should be investigated.

Interestingly, cryogenic electron microscopy studies unveiled that GHRHR SV1 selectively couples to β-arrestins instead of predominantly activating Gs upon GHRH stimulation in HEK293T cells [47]. In this work, the authors showed how a splicing variant strongly drives β-arrestin recruitment despite the canonical Gs signaling pathway. This presents an opportunity for further studies on GPRCs splicing variants, since Cong and co-authors showed how structural alternations not only allowed normal cells to function via a full-length GHRHR but also led to tumoral cell proliferation through a splice variant upon GHRH stimulation, which is the same endogenous ligand.

Additionally, in recent years, a role of miRNAs in regulating GH secretion was unveiled, together with many studies that addressed how they can directly target several SFs, influencing the global alternative splicing profile. Specifically, the role of two specific miRNAs (let-7e and miR-328-5p) in directly targeting GHRHR SVs was pointed out [48]. In accordance, it was reported that let-7e and miR-328-5p miRNAs are able to regulate GH synthesis by targeting the different GHRHR SVs and significantly inhibit GH expression and proliferation of primary pituitary cells derived from pigs [49].

Interestingly, GH has been shown to be differentially regulated by canonical GHRHR or its SVs: GHRHR acts through the NO/NOS signaling pathway, whereas GHRHR SV1/SV2 exert their regulation through the PKA/CREB signaling pathway. This was confirmed by the changes in signaling pathways after transfection of GHRHR SVs expression vectors in GH3 cells [49].

### 4.3. In1-Ghrelin

Ghrelin gene (GHRL) products and associated factors constitute a complex system that has been demonstrated to play a relevant role, especially in regulating pituitary hormonal secretion. Moreover, some components of the ghrelin family have been linked to the development of certain endocrine-related tumors, including PitNETs [50,51].

Over the years, increasing attention has been given to ghrelin, the direct active peptide product of GHRL; in particular, one of its splicing variants, In1-ghrelin, has been associated with the development and aggressiveness of different NETs, e.g., in prostate and breast cancer [52,53,54]. In1-ghrelin variant is generated by retention of intron 1, resulting in an alteration in the amino acid sequence of the C-terminal portion as compared with native ghrelin. However, the In1-ghrelin variant shares the signal peptide and an initial portion of 13 amino acids of its native sequence.

Dysregulation affecting the ghrelin system in PitNETs was unveiled by Vazquez Borrego and co-authors: specifically, the oncogenic splicing variant In1-ghrelin, which is poorly expressed in normal tissue, was highly expressed in both functioning (GH-, ACTH-, PRL-PitNETs) and NF-PitNETs and was associated with increased aggressiveness [13,55] (Table 2). These findings indicate that studying the alteration of the ghrelin system, specifically its In1-ghrelin variant, could contribute to a better understanding of PitNETs pathogenesis. This suggests that the In1-ghrelin variant, among others, would be helpful in providing novel diagnostic and potential therapeutic targets in PitNETs.

### 4.4. SST5TMD4

The absence of SST expression and the identification of different SST isoforms have been extensively studied over the years to find a correlation between SRLs responsiveness and different features of PitNETs.

It is important to state that, in contrast to most GPCRs, SSTs have a unique feature: they are encoded by genes devoid of introns within their coding sequence. However, this does not preclude the generation of spliced variants; in fact, two different splicing variants of SST5 have been unveiled (SST5TMD4 and SST5TMD5), containing four or five transmembrane domains, respectively; thus, they were renamed based on the number of transmembrane domains (TMDs) [56].

The results from different studies have demonstrated that the oncogenic splicing variants SST5TMD4/5, which seem to exhibit subcellular localization [56], are poorly expressed in normal tissue but highly expressed in neuroendocrine tumors (NETs), including PitNETs. Moreover, a study conducted by Luque and co-authors [57] highlighted that truncated SST5 variants are differentially expressed in PitNETs (e.g., SST5TMD4 was expressed in 90% of GH-PitNETs, while it was only expressed in 50% of NF-PitNETs) compared to normal pituitary.

Furthermore, it was shown that SST5TMD4 contributes to oncogenesis, resulting in an increase in malignant features and hindering response to SRLs treatment in GH-secreting PitNETs [57,58]. Specifically, a correlation between SST5TMD4 expression and invasive behavior of GH-PitNETs was found (Table 2).

Indeed, it was demonstrated that SST5TMD4 interacts with SST2 in the intracellular compartment, impairing the proper localization of SST2 at the plasma membrane and preventing its signal transduction [59]. In addition, the dominant-negative function of SST5TMD4 towards SST2 was suggested to contribute to an increase in tumoral cell invasiveness [57,60] by preventing the anti-invasive activity of SST2 in GH-PitNETs [61] (Table 2).

Interestingly, authors have highlighted an inverse correlation between the SST2/SST5TMD4 ratio and the percent decrease in GH and IGF1 levels after postsurgical octreotide-LAR treatment. Additionally, a higher SST2/SST5TMD4 ratio was observed in patients achieving hormonal control (GH and IGF1 levels) after octreotide-LAR therapy compared with uncontrolled patients [57]. Moreover, a correlation between these two truncated variants and SFs was shown. In fact, an association between the mRNA levels of two SFs (SRSF1 and MAGOH) and SST5TMD4 was detected [13]. Specifically, SRSF1 mRNA levels were directly correlated with the aberrant splicing variant SST5TMD4 in NF-PitNETs, GH-, and ACTH-PitNETs, while MAGOH only showed a correlation with the latter [13]. The fact that the spliceosome component SFRSF1 is similarly dysregulated in all PitNET subtypes, correlating also with aberrant splicing variants, despite the high heterogeneity of these tumors, led the authors to speculate about the possible existence of previously unrecognized common driver alterations in PitNETs. This could represent a starting point for the identification of novel therapeutic targets.

### 4.5. DRD2

DRD2 is a GPCR belonging to a family that includes five subtypes of receptors (DRD1-5), expressed in all PitNET subtypes, but mainly in PRL- and NF-PitNETs. The presence of introns within the coding regions of D2-like receptors gives rise to the formation of two different isoforms through alternative splicing, named DRD2 short (D2S) and DRD2 long (D2L) [62]. D2S and D2L differ in the presence or absence of 29 amino acids in the third intracellular loop and display distinct physiological and pharmacological properties, even if in physiological conditions D2L and D2S expression is committed to maintain PRL synthesis and secretion [63,64,65].

D2S and D2L differentially modulate two fundamental pathways in the regulation of lactotroph cell homeostasis: the mitogen-activated protein kinase (MAPK) and the PI3K/AKT pathways. In detail, it was shown that in mouse lactotrophs, D2S activated ERK1/2 and PI3K/AKT pathways, thus causing a reduction in cell proliferation, whereas D2L exerted an inhibitory effect on them [66]. The authors demonstrated that simultaneous knockdown of both D2L and D2S resulted in ERK1/2 inhibition and AKT activation, leading to uncontrolled cell proliferation and consequent pituitary hyperplasia and hyperprolactinemia [66]. This suggests that the D2L/D2S ratio can influence the responsiveness to pharmacological treatment in tumoral lactotrophs. Several studies have tried to find a possible correlation between the expression of DRD2 or its isoforms and DA responsiveness in PRL and NF-PitNETs; however, there are contrasting data in the literature.

In PRL-PitNETs, it has been shown that drug-resistant tumors expressed lower levels of D2S mRNA compared with responsive ones [67,68], although reduced expression of D2L in cabergoline-resistant versus cabergoline-sensitive tumors was also described [69] (Table 2).

Also, the expression of D2S rather than D2L was associated with the most favorable response to DA treatment of NF-PitNETs [70] (Table 2). Accordingly, it was demonstrated that D2S isoform expression in pituitary GH-secreting GH3 cells previously lacking both isoforms improves sensitivity to the DAs bromocriptine action [71]. Furthermore, it was revealed that estrogens can affect the D2S/D2L ratio, promoting an increase in D2L expression and therefore affecting the efficacy of DA treatment in PRL-secreting PitNET, as demonstrated in mouse normal pituitary and pituitary tumor cell lines [72,73,74] (Table 2). Moreover, a study conducted by Oomizu and co-authors provided evidence that estradiol was able to modulate DRD2 alternative splicing, thereby reducing the inhibitory effect of BRC on PRL release in pituitary-derived cells [75].

### 4.6. CCND1

The CCND1 gene may undergo AS to generate two isoforms: the conventional cyclin D1a and the shorter cyclin D1b. The AS event that produces the cyclin D1b transcript arises from a failure to splice at the CCND1 exon 4–intron 4 boundary due to a polymorphism within the exon 4 splice donor site (G/A870), wherein the A allele was suggested to favor cyclin D1b production. The usage of this alternative 5′SS in exon 4 introduces a premature termination codon, resulting in an isoform that lacks the C-terminal protein domain containing both a GSK-3β phosphorylation site and a CRM1-dependent nuclear export domain, and is constitutively expressed in the nuclei [76,77]. Cyclin D1a is a key regulator of cell proliferation, promoting cell cycle G1/S transition, and an increase in its activity has been well established in human tumorigenesis [77], whereas the cyclin D1b isoform displays different oncogenic functions that mostly impact invasion and metastasis processes [76,78,79,80]. Increased expression of CCND1b has been observed in multiple types of cancers, including breast, lung, and prostate cancer [81].

In PitNETs, CCND1 allele frequencies and genotype distribution were examined in a large cohort of patients with sporadic PitNETs of various histology [38].

Within the 294 patients analyzed, 54 had evidence of recurrent PitNETs, which comprised 37 NF-PitNETs, 8 GH-PitNETs, 4 ACTH-PitNETs, and 5 PRL-PitNETs. Interestingly, a progressive increase in the CCND1 AA and a decrease in the distribution of the CCND1 GG genotype from grade 1 through to grade 4 tumors were observed across the whole cohort, suggesting that CCND1 A alleles may affect tumor progression by inducing cyclin D1b transcript production [38] (Table 2). Similarly, the CCND1 AA genotype was more frequently found in thyroid cancer than in benign tumors, and nuclear cyclin D1b expression was associated with more aggressive features in papillary thyroid carcinoma [82]. Also, CCND1b expression, but not CCND1a, is correlated with tumor grade, metastasis, and patient survival in lung and breast cancer [83,84]. It is worth noting that SRSF1, known to be dysregulated in PitNETs as previously mentioned, works as a critical allele-selective factor that is directly associated with the cyclin D1b transcript and promotes cyclin D1b production in cancer cell models [85]. Thus, identification of SRSF1 as a disease-relevant effector of cyclin D1b suggests that suppressing the oncogenic alternative splicing process might represent a novel strategy to limit tumor progression in PitNETs.

### 4.7. Other Genes

Moreover, aberrant transcripts from additional genes are constantly emerging since transcriptome profile characterization of PitNETs has become an active field of research over the last few years. In particular, the NTRK1 splice variant TrkAIII is noteworthy, as its oncogenic role in neuroblastoma cells has been well established [86], and it has been recently detected in invasive PIT-1 positive PitNETs [86]. In PitNETs, TrkAIII is correlated with HIF-2α expression and TrkA phosphorylation. These findings suggest a link between hypoxia response activation and its involvement in tumor pathogenesis and progression, providing a rationale for extending Trk inhibitor therapy in PitNETs [86]. In GH-PitNETs, Huang and co-authors reported abnormal exon inclusion in the Neural Cell Adhesion Molecule 1 (NCAM1) gene as the top splicing event [14] (Table 2). They demonstrated that knocking down the aberrant exon in primary cells from GH subtype patients resulted in significantly suppressed GH secretion, whereas overexpression of the long aberrant transcript promoted GH release, indicating that altered NCAM1 splicing may be linked to the pathogenesis of GH-PitNETs, aligning with previous studies [87].

**Table 2:** Clinically relevant aberrant splicing variants in PitNETs. The alternative splicing event occurring, the structural or protein changes generated, the functional effects in PitNETs, and clinical relevance have been summarized.

Gene	Splicing Variant	Alternative Splicing Event	Structural/Protein Change	Functional Effects	Clinical Relevance	Reference
*ESRRG*	**Aberrant ESRRG**	Cryptic 3′ splice-site in intron 4 induced by SF3B1^R625H^ mutation	Gain-of-function ESRRG with increased interaction with Pit-1 transcriptional cofactor	Enhanced PIT-1 binding; increased PRL transcription and secretion; ER-independent mechanism	Explains hyperprolactinemia in SF3B1-mutant prolactinomas; associated with a higher proportion of male patients	[32]
*GHRHR*	**GHRHR SV1 and SV2**	Multiple splice variants; SV1 replaces exons 1–3 with part of intron 3	First 89 amino acids replaced by a unique 25-aa sequence, truncating the extracellular domain	Regulation of GH secretion through the PKA/CREB pathway	Potential contributor to PitNET proliferation and signaling; possible therapeutic target; regulated by let-7e and miR-328-5p	[49]
*GHRL*	**In1-ghrelin**	Intron 1 retention	Altered C-terminal sequence while preserving the signal peptide and first 13 amino acids	Association with increased tumor aggressiveness	Potential diagnostic biomarker and therapeutic target	[55]
*SST5*	**SST5TMD4**	Non-canonical cryptic intron splicing event within the SST5 transcript	Truncated receptor with 4 transmembrane domains and altered intracellular localization	Interaction with SST2, impairing membrane localization and signaling; increased invasiveness; reduced SRLs response	Marker of aggressive disease and poor response to octreotide-LAR; SST2/SST5TMD4 ratio predicts treatment response	[57,58,59,60,61]
*DRD2*	**D2S/D2L**	Alternative splicing generates short (D2S) and long (D2L) isoforms	Differ by the presence/absence of 29 amino acids in the third intracellular loop	Differential regulation of MAPK and PI3K/AKT pathways	D2S expression correlates with better responsiveness to DAs in PRL- and GH-PitNETs. D2L/D2S ratio could influence the responsiveness to pharmacological treatment in PRL-PitNETs	[67,68,69,70,71,72,73,74,75]
*CCND1*	**Cyclin D1b (CCND1b)**	Exon 4–intron 4 junction splicing failure due to G/A870 polymorphism	Premature stop codon with loss of the C-terminal GSK-3β phosphorylation site and CRM1 nuclear export domain leading to cyclin D1b constitutive expression in the nuclei	Promotes invasion and metastasis	CCND1 AA genotype correlates with tumor progression	[38]
*NTRK1*	**TrkAIII**	Skipping of exons 6, 7 and 9	Loss of amino acids 192–284 from the extracellular D4-Ig-like domain driving TrkAIII ligand-independent intracellular activation	Associated with HIF-2α expression and TrkA phosphorylation, suggesting activation of hypoxia pathways	Potential biomarker for Trk inhibitor therapy	[86]
*NCAM1*	**Long NCAM1 isoform (NCAM1-L)**	Abnormal inclusion of exon 9	Longer intracellular (cytoplasmic) domain of NCAM-1 with enhanced downstream signaling activity	Promotes GH secretion	Implicated in GH-PitNET pathogenesis	[14,87]

## 5. Therapeutic Strategies Targeting Splicing

The increasing knowledge of non-familial cancers expressing alternatively spliced proteins that contribute to malignant phenotypes has prompted research into novel molecules capable of interfering with alternative splicing processes and ultimately exerting antitumoral effects [88]. Currently, three different strategies to target alternative splicing in cancer are under investigation, including the use of core spliceosome inhibitors, targeting of splice modulators, and splice-site modulation.

### 5.1. Spliceosome Inhibitors

A first strategy to block AS is based on the evidence that tumoral cells are characterized by a higher metabolic rate compared to normal cells and consequently have increased splicing rates [89]. These two cell signatures make tumoral cells more suitable for direct splice inhibitors that would not affect normal cells. A clear example is provided by several studies, in which targeting SF3B1 with specific and selective drugs, such as pladienolide-B or its derivatives, demonstrated an inhibition of tumoral cells’ viability and proliferation in different kinds of NETs [13,90,91,92,93,94]. Pladienolide-B is an antitumor macrolide that binds to the HEAT repeat domain of SF3B1 and stabilizes the protein in its open conformation, preventing the transition to the closed conformation needed to form the branch-site adenosine-binding pocket and stably accommodate the BS/U2 duplex [27].

**Figure 1:**
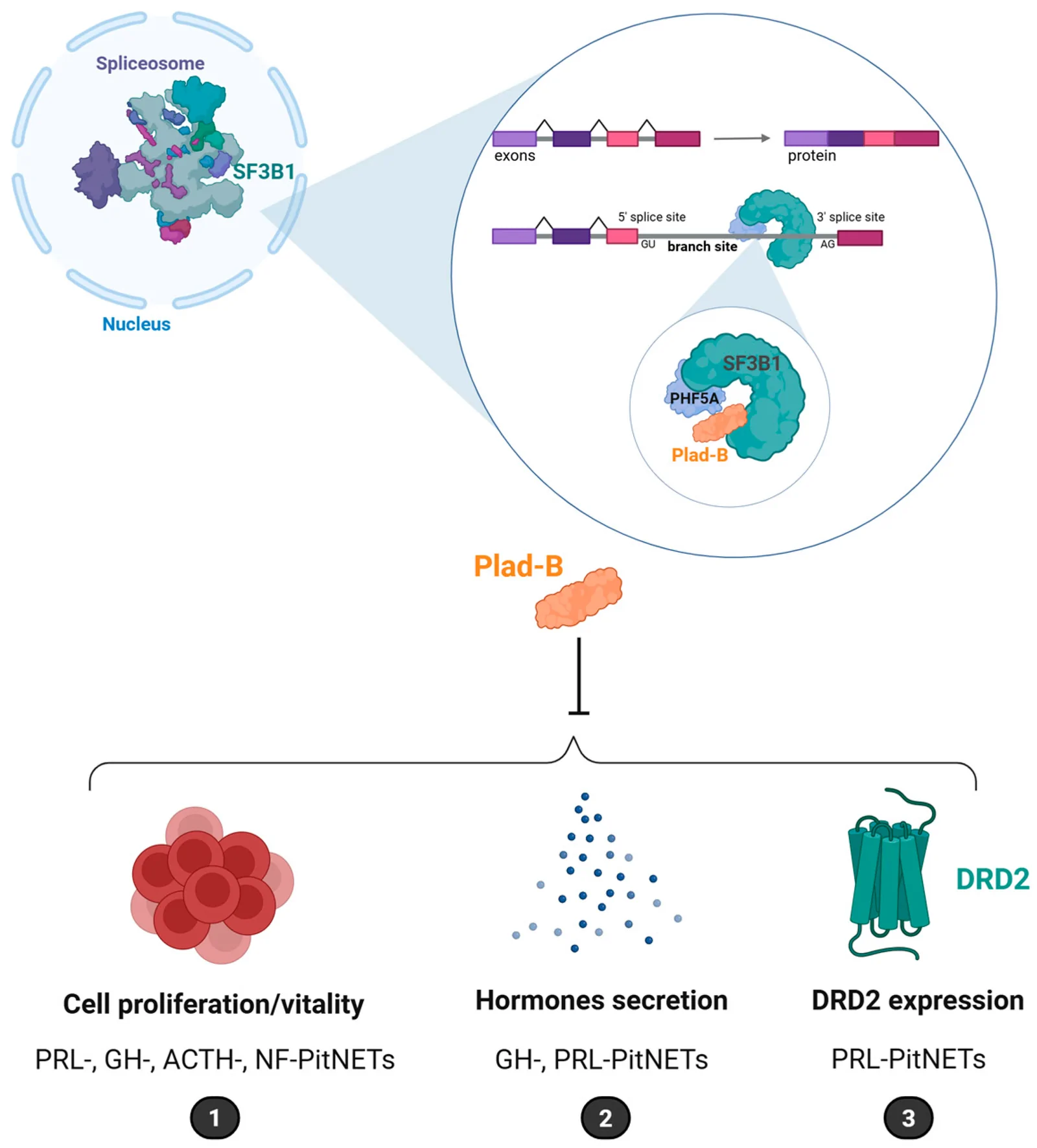
Pladienolide-B’s effects on different PitNET cells’ tumoral endpoints. Pladienolide-B treatment resulted in (**1**) a reduction in cell proliferation in PRL-, GH-, ACTH-, and NF-PitNET cells; (**2**) inhibition of GH and PRL secretion in GH- and PRL-PitNETs, respectively; and (**3**) reduced DRD2 receptor expression in tumoral lactotrophs.

Pladienolide-B exerted antimitotic action in PRL-PitNETs [45] as well as in primary cultured cells from GH-, ACTH-, PitNETs and NF-PitNETs [13]. An inhibitory effect on GH and PRL secretion was also shown [13,37] in primary cultured GH- and PRL-PitNET cells, respectively, as illustrated in Figure 1. The inhibitory effects of pladienolide-B on cell proliferation were shown in GH- and ACTH-secreting cell lines, GH3 and AtT-20, respectively [13]. Moreover, pladienolide-B showed a significant inhibition of cell proliferation, vitality, hormonal secretion, migration, and promoting cell apoptosis in PRL-secreting MMQ cells [37] (Figure 1). In addition, pladienolide-B’s antiproliferative, antisecretory, and antimigratory effects have also been confirmed in MMQ cells overexpressing SF3B1^R625H^ [37], contrary to the DAs cabergoline. Pladienolide-B’s antimitotic, proapoptotic, and antisecretory effects were maintained in primary cultured cells from both DA-resistant and -responsive PRL-PitNETs. Interestingly, pladienolide-B was shown to reduce DRD2 expression at both the transcript and protein levels [37], as shown in Figure 1. Furthermore, it is worth noting that two different clinical trials with the pladienolide-B derivative E7107 have been conducted in patients with solid tumors (NCT00499499 and NCT00459823) [95,96]. Mechanistically, E7107 acts as pladienolide-B by preventing U2 snRNP binding to the pre-mRNA branch point. The results of the first-in-human, phase I open-label, single-arm clinical trial (ClinicalTrials.gov ID: NCT00499499), in which the maximum tolerated dose, dose-limiting toxicities, and pharmacokinetic profile of E7107 were determined, have been reported [95].

In detail, two patients experienced dose-limiting toxicities including diarrhea, vomiting, dehydration, and myocardial infarction on days 1 and 3 after administration of the maximum E7107 dose (5.7 mg/m^2^), leading to 4.3 mg/m^2^ being the maximum tolerated dose. The most common drug-related adverse events were nausea, vomiting, and diarrhea. Vision loss was experienced by two patients, which put the study on clinical hold, leading to the suspension of the clinical trial. Pharmacokinetic analysis showed that E7107 was rapidly distributed, with a moderate elimination half-life (6–13 h) and high clearance, and that drug exposure increased in a dose-dependent manner [95]. Pharmacodynamic analysis revealed a dose-dependent and reversible inhibition of pre-mRNA processing of target genes, confirming proof-of-principle activity of E7107 [96]. Based on these results, E7107 is considered a unique first-in-class molecule.

Certainly, the development and testing of new small molecules capable of inhibiting the spliceosome, with minimal side effects and a better safety profile, will be a challenging therapeutic opportunity.

### 5.2. Targeting of Splicing Modulators

The manipulation of splice modulators, such as SRSFs, represents an indirect approach to modulating splicing activity. ATP-competitive inhibitors of SRPK and CLK kinases have been discovered in large-scale screening efforts. These compounds limit SRSF phosphorylation, ultimately affecting the splicing profile of several genes. Among SRPK kinase inhibitors, SRPIN340 inhibits both SRPK1 and SRPK2, whereas the most recently developed SPHINX31 selectively inhibits SRPK1 [97,98,99]. Their most characterized anti-tumoral effect is explicated through a reduction in SRSF1 phosphorylation and activity, which leads to a decrease in VEGF splicing isoform production (specifically the VEGF165a isoform, referred to as VEGF164a in rodents) and, in some cases, a switch in VEGF splicing towards its anti-angiogenic splice variant (VEGF165b) [100,101]. This results in angiogenesis inhibition and tumor growth retardation, as also demonstrated in in vivo models of prostate and colorectal cancer [97,99].

**Figure 2:**
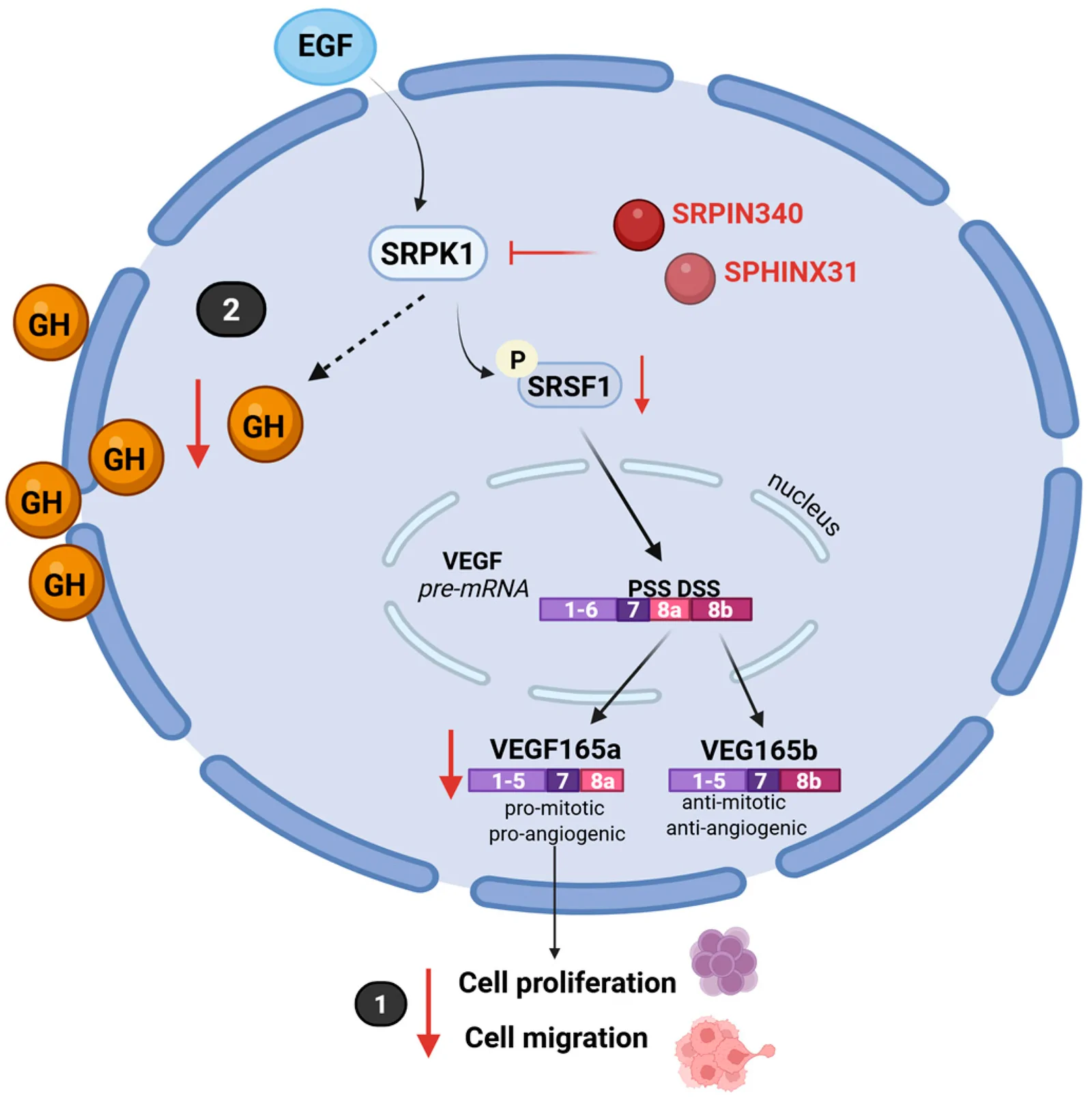
Inhibition of SRPK1 effects in GH-PitNET cells. (1) SRPK1 inhibitors SRPIN340 and SPHINX31 reduced SRSF1 phosphorylation levels, leading to a decrease in proximal splicing site (PSS) selection in the VEGF gene. This resulted in a reduction in the pro-mitotic and pro-angiogenic VEFG-A165a isoform production, inhibiting GH4C1 cell proliferation and migration. (2) SRPK1 inhibition by SRPIN340 reduced GH levels in primary cultured cells from GH-PitNETs. Red arrows indicate the direct effects exerted by SRPIN340 and SPHINX31 treatment.

A recent study from our group has shed new light on the antitumoral properties of SRPK inhibitors in GH-PitNET cells [102], as shown in Figure 2. Briefly, in GH4C1 cells, both SRPIN340 and SPHINX31 were able to exert cytostatic and cytotoxic effects and inhibit cell migration. These effects were related to a downstream decrease in SRSF1-induced VEGF164a pro-survival isoform production (Figure 2). Moreover, SRPIN340 was capable of reducing GH secretion in GH-PitNETs independently of their in vitro responsiveness to octreotide, suggesting that SRPK1 inhibitors may represent a novel strategy to treat SRL-unresponsive GH-PitNETs [102]. Although large-scale studies are needed, there are pre-clinical data indicating that VEGF is a potential therapeutic target in PitNETs. Anti-VEGF therapies have shown potential in treating refractory PitNETs and pituitary carcinomas resistant to conventional treatments [103]. In this scenario, modulating VEGF alternative splicing by small-molecule SRPK1 inhibitors could help overcome the side effects of antibody-based therapy [104]. Nevertheless, further studies assessing drug validation and safety of SRPK1 inhibitors certainly need to be conducted.

CLK inhibitors have also been developed, some of which are currently under investigation in a phase I clinical trial in advanced solid tumors. For example, an open-label, multi-center, dose-escalation study on the SM08502 inhibitor, including its pharmacokinetics and safety, has been conducted (ClinicalTrials.gov ID: NCT03355066). Furthermore, promising in vivo data from a study conducted by Iwai and co-authors showed that the CLK inhibitor T-025 can induce breast cancer cell death through alternative splicing modulation of the MYC gene, which is known to be involved in RNA splicing, RNA transport, cell cycle, and DNA repair pathways [105]. The authors’ results suggest that the novel CLK inhibitor could have therapeutic benefits, especially in MYC-driven cancer patients; however, more in-depth studies are needed.

Since there are currently no studies exploring their effects in PitNET cells, these findings represent a starting point for future research.

### 5.3. Splice-Switching Oligonucleotides: Future Perspective for PitNET Therapy

To directly modulate alternative splicing that contributes to malignant phenotypes, a promising strategy could involve targeting pre-mRNA splicing sites responsible for the generation of aberrant transcripts through RNA-based approaches. This is a splicing-correcting therapy based on the use of antisense oligonucleotides (ASOs), which are chemically modified RNA molecules 15–30 nucleotides in length; their specificity comes from their complementary binding to unique sequences on mRNA, allowing correction of only the targeted spliced isoform. ASO-mediated redirection of cancer-associated splicing isoforms has already been demonstrated both in vitro in different cell lines and in vivo in xenograft tumor models [8]. Nevertheless, efficient delivery to specific target organs and intracellular compartments remains a challenge for their use in clinical practice [106].

Interestingly, ASOs can act as splicing modulators, termed splice-switching oligonucleotides (SSOs), which mask critical splicing sites, causing either exon/intron skipping or retention, thus resulting in altered protein structures or the production of different isoforms [107]. The efficacy of SSOs has already been demonstrated in other pathologies and could represent an interesting novel approach to broaden the therapeutic options for heterogeneous pathologies, such as PitNETs.

## 6. Conclusions and Future Directions

To conclude, this review summarized the role of splicing machinery dysregulation in PitNETs, emphasizing its role in the tumorigenesis of all PitNET subtypes and providing a potentially rich source of novel therapeutic targets for alternative pharmacological approaches to treating resistant or aggressive tumors.

Specifically, different RBPs linked to EMT, chiasmatic compression, or invasiveness, such as ESRP1, RAVER1, and MAGOH, have been shown to be altered in PitNETs.

For instance, the identification of variants in splicing factors, such as SF3B1, in PRL-PitNETs has unveiled the potential role of AS in promoting PitNETs aggressiveness and affecting pharmacological responsiveness.

Additionally, splicing variants in genes such as DRD2, SST5, ESRRG, GHRHR, In1-ghrelin, and CCND1, which regulate different pathways involved in PitNET tumorigenesis and resistance, have been described.

The studies that have been reported represent a fundamental initial step in highlighting the significant role of splicing in shaping PitNET heterogeneity. Above all, the valuable insights provided by transcriptomic analyses have not only improved the understanding of PitNET classification and diagnosis but have also suggested novel directions for optimizing clinical treatment strategies and prognostic predictions, especially for challenging PitNET subtypes, such as silent corticotrophs.

It is also worth mentioning that the full splicing landscape of PitNETs and the molecular mechanisms underlying the changes in SFs remain largely unexplored, since differences at the single-cell level have not been optimized yet. Thus, it is important to develop new methodologies for single-cell splicing discovery.

The identification of other SFs commonly dysregulated in different PitNET subtypes could pave the way toward the identification of novel therapeutic targets based on the dysregulation of these key elements. The development of specific drugs, such as pladienolide-B or its derivatives, capable of modulating the splicing process and exerting antitumor effects in PitNETs, is challenging. However, further pre-clinical studies evaluating their safety and adverse effects should be conducted to support the development of novel therapies.

Furthermore, advances in RNA-based therapeutics, such as SSOs capable of modifying splicing, could represent a promising novel approach to expanding the clinical therapeutic options for PitNETs.

To conclude, the integration of these splicing features could drive molecular classification and treatment approaches for different PitNET subtypes, thereby improving clinical outcomes.

## Data Availability

No new data were created or analyzed in this study. Data sharing is not applicable to this article.
